# Impact of human activities on subaqueous topographic change in Lingding Bay of the Pearl River estuary, China, during 1955–2013

**DOI:** 10.1038/srep37742

**Published:** 2016-11-25

**Authors:** Z. Y. Wu, Yoshiki Saito, D. N. Zhao, J. Q. Zhou, Z. Y. Cao, S. J. Li, J. H. Shang, Y. Y. Liang

**Affiliations:** 1 Key Laboratory of Submarine Geosciences and Second Institute Oceanography, State Oceanic Administration, Hangzhou 310012, China; 2 School of Earth Sciences, Zhejiang University, Hangzhou 310027, China; 3 Geological Survey of Japan, AIST, Central 7, Higashi 1-1-1, Tsukuba, Ibaraki 305-8567, Japan; 4 ReCCLE, Shimane University, 1060, Nishikawatsu-Cho, Matsue 690-8504, Japan

## Abstract

Estuaries have been sites of intensive human activities during the past century. Tracing the evolution of subaqueous topography in estuaries on a decadal timescale enables us to understand the effects of human activities on estuaries. Bathymetric data from 1955 to 2010 show that land reclamation decreased the subaqueous area of Lingding Bay, in the Pearl River estuary, by ~170 km^2^ and decreased its water volume by 615 × 10^6^ m^3^, representing a net decrease of 11.2 × 10^6 ^m^3^ per year and indicating the deposition of approximately 14.5 Mt/yr of sediment in Lingding Bay during that period. Whereas Lingding Bay was mainly governed by natural processes with slight net deposition before 1980, subsequent dredging and large port engineering projects changed the subaqueous topography of the bay by shallowing its shoals and deepening its troughs. Between 2012 and 2013, continuous dredging and a surge of sand excavation resulted in local changes in water depth of ± 5 m/yr, far exceeding the magnitude of natural topographic evolution in Lingding Bay. Reclamation, dredging, and navigation-channel projects removed 8.4 Mt/yr of sediment from Lingding Bay, representing 29% of the sediment input to the bay, and these activities have increased recently.

Human activities have important impacts on rivers and their deltas. On a global scale, rivers transport 36,000 km^3^ of freshwater and ~20,000 Mt of sediment to the ocean each year, providing freshwater, nutrients, transportation and human sustenance^1^,^2^,^3^,^4^,^5^. The transport of sediment by rivers to the ocean is not only an important part of global material cycles but also an important indicator of land degradation and environmental change^6^,^7^,^8^,^9^,^10^. The seaward edge of a delta is an important interface for material exchanges between land and sea, and it plays a dual role as a driver and recorder of natural and anthropogenic environmental changes^11^.

The five great rivers of East and Southeast Asia—the Yellow (Huanghe), Yangtze (Changjiang), Pearl (Zhujiang), Red (Song Hong), and Mekong—are key regions for studies of the impact of human activities on deltas. As these five rivers have been increasingly affected by damming, the amount of terrigenous material transported from this region to the western Pacific has dropped from ~2,000 Mt/yr to ~600 Mt/yr over the last millennium^6^. This decrease has affected the Yangtze River delta by allowing erosion at its delta front^12^. The Yellow River delta, because of river channel shifting and the implementation by China of its water-sediment regulation scheme (WSRS), has expanded into the ocean since the beginning of this century^13^,^14^,^15^.

The Pearl River, one of the 25 largest rivers in the world and the third-largest river in China in terms of water discharge, has also been affected by human activities. This study sought to determine the recent history of natural and human processes occurring in its delta.

The Pearl River delta is distinctive in that it combines large volumes of water in a plexus of closely spaced distributary streams with small amounts of sediment^16^,^17^,^18^ (Fig. 1). Human activities have substantially changed the coastline and bathymetry in the Pearl River delta^19^. More than 14,000 reservoirs have been built in the Pearl River drainage basin, trapping large amounts of sediment and causing a drastic decrease in sediment transport^20^,^21^,^22^,^23^,^24^. About 90% of the sediment in the Pearl River historically came from its main branch, the West River, but this sediment input declined abruptly after completion of the Yantan Reservoir in 1992 and the Longtan Reservoir in 2007. As of 2011, the annual sediment transport into the Pearl River (as measured at the Gaoyao, Shijiao, and Boluo hydrological stations; Fig. 1A) had decreased nearly sixfold to 12.7 Mt/yr, or only 17.7% of the average of 71.6 Mt/yr from 1950 to 2011^25^ (Fig. 2A). How has the subaqueous delta topography in the Pearl River responded?

Massive, unauthorized sand excavation, which began in the 1980 s and peaked in the 1990 s, in the Pearl River network is another important human activity affecting the regional topography. The total volume of sand excavated from 324 segments of the Pearl River network from 1986 to 2003 exceeded 870 × 10^6^ m^3^, whereas annual deposition of sand during this period was only 8–10 × 10^6^ m^3^/yr^26^. Thus, 17 years of excavation displaced a volume representing a century of deposition. Deepening of the riverbed has significantly changed the divided flow ratio (DFR) of the eight distributaries of the Pearl River (Fig. 1B). The DFR of the North River, from Sixianjiao to the confluence with the West River, has increased by 8.8%, and the DFR of the four eastern distributaries into Lingding Bay, the funnel-shaped estuary that constitutes the north-eastern part of the subaqueous Pearl River delta, has increased by 7.7%^26^. The proportions of water and sediment discharge entering these distributaries has changed along with the change of DFR since the 1980s^27^. The water and sediment entering Lingding Bay has increased by 40.5 × 10^9^ m^3^/yr and 2.95 Mt/yr, respectively, or the equivalent of the entire output of the North River^28^.

Lingding Bay is the sole access to the sea for the major ports of Guangzhou and Shenzhen. The main navigation channel is dredged every year, and new channels are also being dredged. Dredging deepens the troughs of the bay, and deposition of the sand spoil makes the area surrounding the troughs shallower, accentuating the existing topography in the combination expressed as “shallower shoals, deeper troughs’’^27^,^29^. Furthermore, sand excavation and large-scale land reclamation are ongoing throughout Lingding Bay.

In this study we compiled 55 years of bathymetry and shoreline data along with newly collected bathymetric data from 2012 and 2013. Here we present these data and discuss the relationships between these changes and human activities in the drainage basin to the bay during the period 1955–2013.

## Results

The land, tidal flats, and subaqueous areas in the study area have changed significantly since the 1950 s. The area of land increased by more than 230 km^2^ from 1955 to 2010 (Table 1, Fig. 3C), and intertidal and subaqueous areas decreased. The change was not gradual and occurred abruptly between 1980 and 2000; therefore, in this section we discuss the bathymetric changes for the periods 1955–1980, 1980–2010, and 2012–2013 separately, and then summarize the changes in water area and volume.

### Bathymetric changes between 1955 and 1980

The bathymetric maps of 1955, 1964, and 1980 show that the basic configuration of two deep troughs and three shoals in Lingding Bay did not change between 1955 and 1980 (Fig. 3A1 and A2). The deepest trough (>20 m) in the Humen channel, which was shown as a single trough in 1955, was divided into separate northern and southern parts on the 1964 and 1980 maps. The average water depth in the study area declined from 4.4 m in 1955 to 4.25 m in 1964 to 4.20 m in 1980 (Table 1). The subaqueous area shrank from 59.3% of the study area in 1955 to 55.4% in 1964 to 53.4% in 1980.

The annual bathymetry change rate (BCR) maps during the two periods 1955–1964 and 1964–1980 (Fig. 3B1 and B2) illustrate the natural evolution of the subaqueous topography in the study area. There were few areas of reclamation, and we found no significant evidence of large-scale human activities in the estuary. The rate of deposition was high at the four Pearl River outlets. Topographic changes away from the outlets indicate a patchy distribution of deposition and erosion, without signs of being related to human activities.

From 1955 to 1964, the area of erosion was 14.4% of the study area and the area of deposition was 39.5% (Fig. 3B1). On average, the BCR was −4.59 cm/yr in the erosion area and 6.48 cm/yr in the deposition area (Table 2). Thus, deposition dominated the topographic changes in the bay, resulting in a decrease of mean water depth.

From 1964 to 1980, the area of erosion increased to 23.2% and the area of deposition decreased to 28.8% of the study area (Fig. 3B2). The average BCR was −3.10 cm/yr in the erosion zone and 4.70 cm/yr in the deposition zone. This indicates a gradual transition from a pattern dominated by deposition during 1955–1964 to a pattern of balance between erosion and deposition. The mean water depth decreased slightly in this period.

### Bathymetric changes between 1980 and 2010

**Figure 1:**
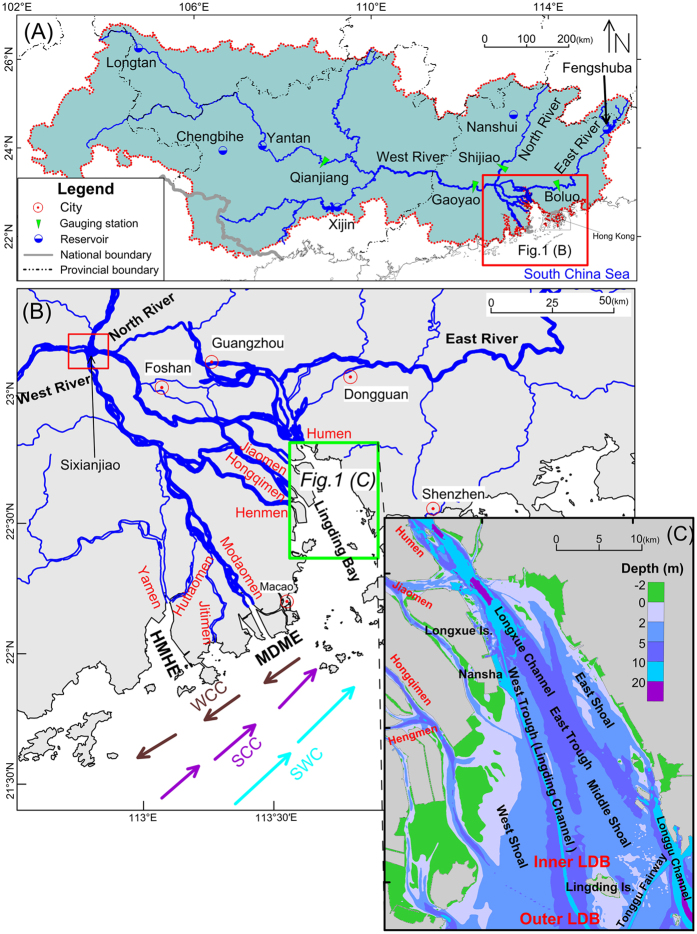
Location maps. (**A**) Pearl River drainage basin. (**B**) The Pearl River network and three sub-estuaries: Lingding Bay, HuangMaoHai esturary (HMHE), and MoDaoMen estuary. (**C**) Detailed map showing the Lingding Bay study area. LDB, Lingding Bay; WCC, Winter Coastal Current; SCC, Summer Coastal Current; SWC, South China Sea Warm Current. Maps created with Surfer™, Version 7, http://www.goldensoftware.com/products/surfer, ArcGIS™, Version 9.2, http://www.esri.com/software/arcgis/arcgis-for-desktop.

From 1980 to 2010, the topography of Lingding Bay remained basically consistent, but evidence of human activities increased markedly. By 2000, the Humen Channel had become much deeper, locally reaching over 20 m (Fig. 3A4). The proportion of subaqueous area (see Fig. 1C) decreased from 53.4% of the study area in 1980 to 49.5% in 2010 (Table 1). After decreasing gradually from 1955 to 1980, the mean water depth of the bay increased to 4.5 m as of 2000. By 2010, the subaqueous area was reduced further to 48.2% of the study area, and the mean water depth had increased to 4.6 m (Table 1).

The BCR maps for the two periods 1980–2000 and 2000–2010 (Fig. 3B3 and B4) showed an increase in land reclamation and deepening of the Lingding Channel due to dredging. The BCR map for 2000–2010 showed growth in areas of deep water at the port of Nansha caused by port construction and along the Middle Shoal caused by dredging of a new waterway, Tonggu Fairway (see Fig. 1C). A large area of erosion was created by dredging in the north, and areas of aggradation were created on both sides of the Tonggu Fairway by dumping of excavated sand around the channel.

From 1980 to 2000, the area of erosion shrank to 20.3% and the area of deposition decreased to 26.7% of the study area. The average BCR was −6.42 cm/yr in the erosion zone and 4.80 cm/yr in the deposition zone. The balance between erosion and deposition changed little during this period compared to 1964–1980; however, the increase in deep areas from new channels resulted in an increase in mean water depth from 4.20 to 4.50 m (Table 1).

From 2000 to 2010, the area of erosion shrank to 14.4% while the area of deposition grew to 31.4% of the study area. The average BCR was −16.0 cm/yr in the erosion zone and 9.35 cm/yr in the deposition zone. The subaqueous topography reflected dramatic changes in erosion intensity and deposition toward “shallower shoals, deeper troughs” that were largely the result of extensive dredging and dumping throughout the Pearl River estuary.

The 5-m and 10-m isobaths showed clear changes in the subaqueous topography since 1955 (Fig. 4A). Although the shape of the 5-m isobath changed from 1955 to 2010, its overall pattern did not change greatly (Fig. 4A1). The shape of the 10-m isobath was similar in 1955, 1964, and 1980 (Fig. 4A2); however, in the 2000 and 2010 maps it changed in response to dredging of existing channels and excavation of new ones (Fig. 4A3 and A4).

### Bathymetric changes between 2012 and 2013

Between 2012 and 2013, the depths changed along 14 of the 20 survey lines in inner Lingding Bay, the result of intensive human activities; however, there were no significant changes in the 13 survey lines in outer Lingding Bay (Fig. 5A).

In inner Lingding Bay, the single-beam echo sounding profiles showed changes in submarine topography of ± 5 m/yr (Fig. 5B1). Widespread annual dredging was conducted near waterways and ports. Sand excavation on the Middle Shoal deepened the seabed there by more than 10 m and resulted in undulations measuring ± 2 m in the seafloor topography (Fig. 5B2). The undulations had wavelengths of 10–70 m and amplitudes of 2–3 m. Sand waves have been reported in this area^30^.

Considerable natural accumulation, more than 2 m/yr in some areas, occurred in the southern part of the Tonggu Channel (Fig. 5B3) after dredging during 2000–2010. The echo-sounding profiles also clearly showed evidence of the second phase of dredging in the Tonggu Channel area.

### Changes of water area and water volume

The bathymetric data indicate continuous decreases in water area and water volume of the study area from 1955 to 2010 (Fig. 3D). During 1955–1964, the water volume was greatly reduced, although there was little change in the land area. Much of the sediment input from the Pearl River into the estuary led to an increase in the area of tidal flats and a decrease in the volume of the estuary (Table 1). During 1980–2010, the subaqueous area declined; however, the water volume was relatively stable because the mean water depth increased owing to dredging in navigational channels. The area with water depths less than 9 m decreased and the area with water depths greater than 10 m increased after 1980, particularly between 2000 and 2010 (Fig. 4B and Table 1).

## Discussion

The bathymetric data show clearly that the subaqueous depositional system of Lingding Bay changed between 1980 and 2000. Other evidence has shown that land reclamation in Lingding Bay amounted to about 6 km^2^ from 1949 to 1987, increased to 356 km^2^ from 1988 to 1997, then almost ceased after 1997^31^. The area shallower than 5 m decreased rapidly during 1980–2000, particularly the area shallower than 2 m (Fig. 4B). Land reclamation was typically done only in areas shallower than about 0.5 m^31^, and it may be that sediment used for reclamation was taken from areas shallower than 5 m.

Another obvious change was the use of dredging for construction of navigation channels and sand mining. The changes in the 10-m depth contour (Fig. 4A3) and the hypsometric curves in Fig. 4B document increases in the areas with water depths of 9–11 m and 18–22 m during 1980–2000 and depths of 9–17 m during 2000–2010. The Tonggu Channel was dug between 2004 and 2007^32^ in connection with port and wharf construction^33^. This channel is clearly visible in Fig. 5B3.

The growth in areas with increasing depth during 2000–2010 (Fig. 3B4) was linked to sand mining. The echo-sounder surveys also showed the same trend in 2012–2013 (Fig. 5B3). Dumping of sand excavated from new channels in nearby shallow areas gave rise to the “shallower shoals, deeper troughs” configuration of today. Since the 1990 s, sand removal for dredging and navigation maintenance has averaged ~19.5 × 10^6^ m^3^ per year^26^,^27^. The area of sand mining has continued to expand^26^,^34^.

Throughout the period 1955–2010, both deposition and erosion have occurred in Lingding Bay. However, deposition was dominant overall, and the area with net deposition was greatest from 1955 to 1964, reaching 430 km^2^.

**Figure 2:**
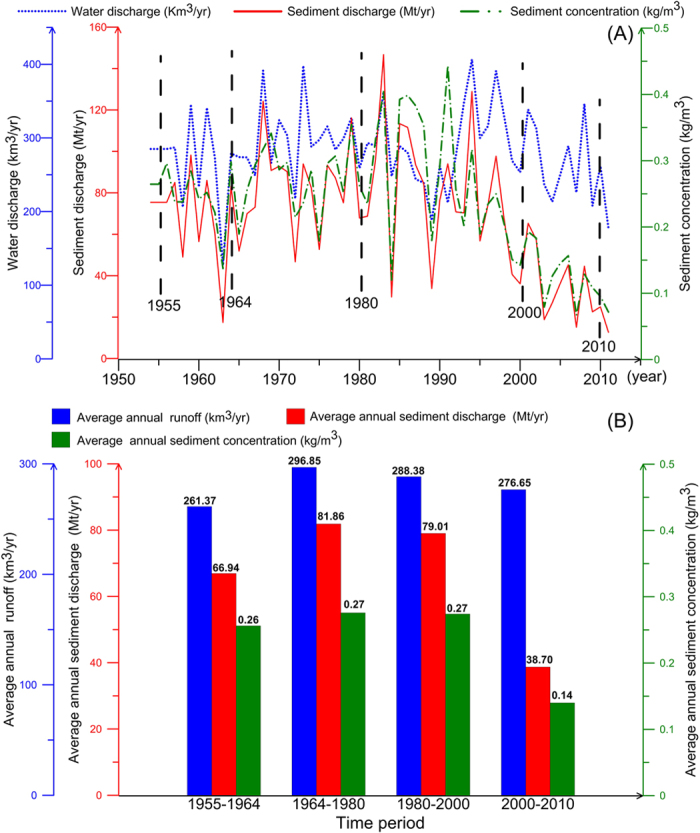
Water and sediment discharge of the Pearl River (sum of the Boluo, Gaoyao, and Shijiao stations). Histograms and line charts created with WPS Office™, Version 2016, http://www.wps.cn/.

The average water depth in Lingding Bay gradually decreased from 1955 to 1980, indicating that the entire estuary was gradually filling with sediment. However, from 1980 to 2010 the average water depth of Lingding Bay gradually increased in response to large-scale reclamation in the estuary in the 1990 s, declining sediment discharge, and dredging. In particular, the completion of the Yantan Reservoir in 1992 and the Longtan Reservoir in 2007 led to a large decrease in sediment transport^20^,^25^. In the period 1955–2000, the average sediment transport of the Pearl River was ~70 Mt/yr. However, from 2000 to 2010, the average was only 38.7 Mt/yr, a decrease of 45% (Fig. 2). After the 1980 s, with increased sand excavation in the watercourse, the DFR of the North River network from Sixianjiao through the West River increased by 8.8%, and the DFR of the four eastern outlets increased by 7.7%^26^. Together, the drastic decrease in sediment input and the increase in sedimentation led by changes of the DFR complicated the sediment discharge into Lingding Bay. The fluctuation in DFR delayed the impact of declining sediment transport on water depths in the estuary. Without the additional sediment discharge into Lingding Bay resulting from the increased DFR, the average water depth would have increased even more. Together, the sharp decrease in the areas of tidal flats and water and the increase in land area (Table 1) shrank the area of shallower water, leading to an increased proportion of deeper water area. The result was that the average depth of Lingding Bay gradually increased.

**Figure 3:**
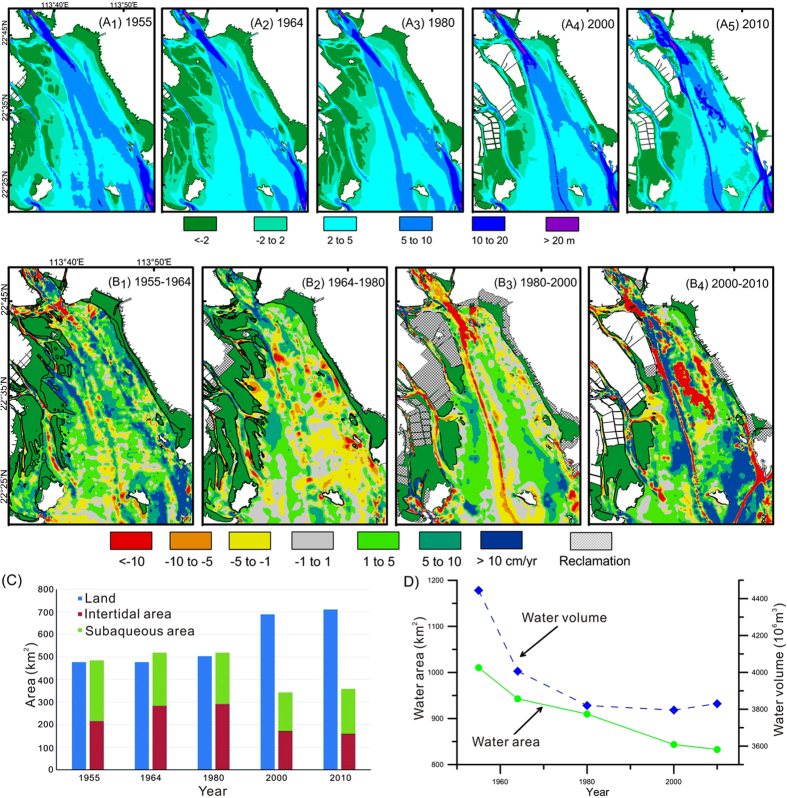
Bathymetric maps of the study area in 1955, 1964, 1980, 2000, and 2010 (**A**) and bathymetry change rate (BCR) maps of the study area during four time periods (**B**). Changes of land area, tidal flat, and water area from 1955 to 2010 (**C**). Total area and volume of Lingding Bay from 1955 to 2010 (**D**). Maps created with Surfer™, Version 7, http://www.goldensoftware.com/products/surfer, ArcGIS™, Version 9.2, http://www.esri.com/software/arcgis/arcgis-for-desktop. Histograms and line charts created WPS Office™, Version 2016, http://www.wps.cn/.

**Figure 4:**
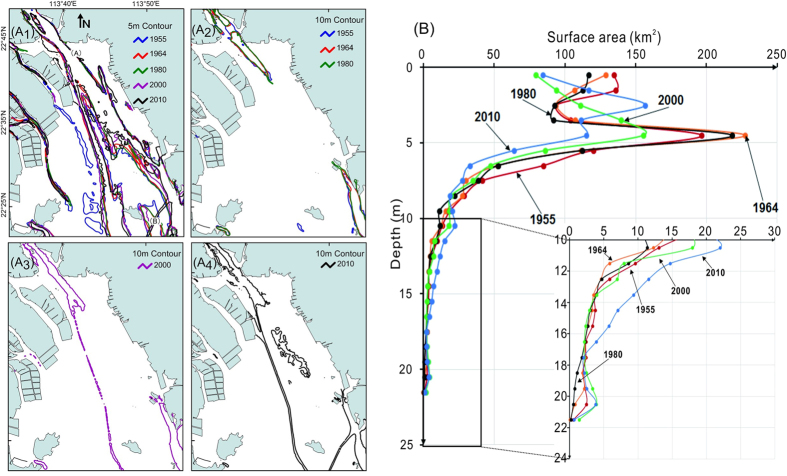
Bathymetric changes in Lingding Bay, 1955–2010 (A_1_–A_4_). Diagram showing subaqueous area of 1-m interval in Lingding Bay from 1955 to 2010. Dredging markedly increased the areas of water deeper than 10 m and shallower than 5 m (**B**). Maps created with Surfer™, Version 7, http://www.goldensoftware.com/products/surfer, ArcGIS™, Version 9.2, http://www.esri.com/software/arcgis/arcgis-for-desktop. Histograms and line charts created WPS Office™, Version 2016, http://www.wps.cn/.

**Figure 5:**
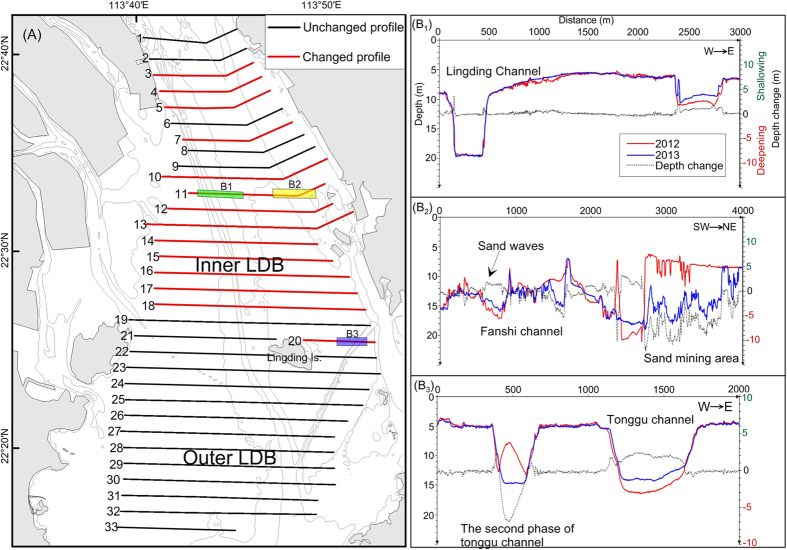
Tracklines of single-beam bathymetric profiles in the study area and depth changes between July 2012 and June 2013 (**A**), and bathymetric changes of profile 11 and profile 20 between 2012 (red) and 2013 (blue). Single-beam bathymetric data was processed by Hypack™, Version 2012, http://www.hypack.com/new/. Maps created with Surfer™, Version 7, http://www.goldensoftware.com/products/surfer, ArcGIS™, Version 9.2, http://www.esri.com/software/arcgis/arcgis-for-desktop. Line charts created WPS Office™, Version 2016, http://www.wps.cn/.

Other studies have shown that of the annual sediment discharge of the Pearl River of ~70 Mt/yr during 1955–2010, 14 Mt/yr was deposited in the Pearl River basin^35^ and 28.6 Mt/yr and 27.4 Mt/yr flowed into the eastern four outlets and the western four outlets^36^, respectively. Of the 28.6 Mt/yr flowing into Lingding Bay, 5.7 Mt/yr (20%) exited the bay to the sea and 22.9 Mt/yr remained in the bay^37^. Our study found that the total change in the water volume of Lingding Bay was 615.09 × 10^6 ^m^3^ during 1955–2010, or 11.18 × 10^6 ^m^3^/yr, which represents an annual sediment accumulation, assuming a dry bulk density of 1.3 g/cm^3^, of 14.54 Mt/yr. This is 8.36 Mt/yr less than the 22.9 Mt/yr of sediment deposition in Lingding Bay estimated by previous studies. What accounts for the difference? As illustrated in Figs 3, 4 and 5, large-scale human activities after the 1980 s, especially since 2000, need to be included in these estimates. To help ensure the sustainability of Lingding Bay, we have to better understand the natural system functioning in the bay.

The Longtan and Yantan reservoirs have sequestered large amounts of sediment from the Pearl River such that its sediment discharge has decreased from ~70 Mt/yr during 1955–2000 to 38.7 Mt/yr during 2000–2010 (Fig. 2). The annual average sediment input to Lingding Bay has decreased from 22.9 Mt/yr to ~12.4 Mt/yr during this time, a reduction of 10.5 Mt/yr; thus, the total sediment input to Lingding Bay decreased by ~105 Mt over a 10-year period. At the same time, the volume of Lingding Bay gradually decreased by siltation in 1955–2000, then increased dramatically during 2000–2010 from the combination of reduced sediment input and sediment removal by dredging in navigation channels and sand excavation (Table 1).

The evolution of subaqueous topography from 1955 to 2010 can be divided into three periods. (1) Before 1980, Lingding Bay evolved naturally by deposition of sediment from the Pearl River and human impacts were negligible. During this period, the area of land in the Lingding Bay study area increased by 25.5 km^2^, and the area of tidal flats increased by 75 km^2^, mostly at the expense of subaqueous area (Table 1). The volume of water in the bay decreased by 625 × 10^6 ^m^3^ and the mean water depth decreased. (2) From 1980 to 2000, land reclamation led to a sharp increase of 186 km^2^ in land area, and tidal flats decreased by 120 km^2^ (Table 1). Although the area covered by water decreased by 66 km^2^, the effect on the total water volume was mostly offset by the construction of navigation channels, and the mean water depth increased. (3) After 2000, excavation activities increased in the distributary network and large-scale sand excavation occurred in the north-central part of Lingding Bay. During this period, the area of land slightly increased by 22.1 km^2^, the area of tidal flat decreased by 11.6 km^2^, and the volume of water increased slightly by 34 × 10^6 ^m^3^ (Table 1).

## Conclusion

From 1955 to 2010, human activities had a substantial effect on sediment discharge into the Pearl River estuary. The average annual flow fluctuated by about ± 6% within each of the periods 1955–1964, 1964–1980, 1980–2000, and 2000–2010, whereas the sediment discharge dropped by 42%.

The subaqueous topography of Lingding Bay was strongly affected by human activities from 1955 to 2010. As a result of land reclamation, the area covered by water in the Lingding Bay study area decreased from 1010 km^2^ to 833 km^2^, and the area of tidal flat decreased from 215 km^2^ to 159 km^2^ over this period. From 1955 to 1980, human activities had a small effect on topography in Lingding Bay. Sediment deposition from the Pearl River decreased the mean water depth of the bay from 4.4 m to 4.2 m. From 1980 to 2010, dam construction greatly reduced the input of sediment at the same time the areas of tidal flat and shallow water decreased dramatically owing to land reclamation. The proportion of shallow-water areas decreased as that of deep water areas increased, and the average water depth increased from 4.2 m to 4.6 m. The impacts of human activity are obvious in the distribution of bathymetry change rates in this period. Bathymetric maps show large dredged channels, widespread dumping areas, and regions of recent sand excavation.

From 1955 to 2010, the water volume in Lingding Bay decreased by 615 × 10^6 ^m^3^, or 9.7 × 10^6^ m^3^/yr. Human activities removed 8.4 Mt/yr of sediment from the estuary, accounting for 29% of the deposition in Lingding Bay, and these activities gradually increased.

Bathymetric surveys in 2012 and 2013 indicated that sand excavation, channel dredging, and excavation of new channels in inner Lingding Bay far exceeded natural processes in their impact on the bay’s bottom topography. Siltation in some artificial channels reached 2 m/yr, posing a serious potential problem for seagoing commerce in this area.

## General setting

### Geography, topography, and bathymetry

The Pearl (Zhujiang) River consists of a drainage basin containing the West, North, and East rivers, and a delta at its end with an area of 8,600 km^2^ (Fig. 1). Its distributary system consists of the confluence of these three rivers in a plexus of deltaic streams that resolves into eight distributaries debouching into the South China Sea through four western outlets (Yamen, Hutiaomen, Jitimen, and Modaomen) and four eastern outlets (Hengmen, Hongqimen, Jiaomen, and Humen) (Fig. 1B). The Pearl River delta has three major estuaries: the Huangmaohai estuary for the Yamen and Hutiaomen distributary, the Modaomen estuary for the Modaomen distributary, and the Lingding Bay estuary (called Lingdingyang in Chinese) for the four eastern distributaries. The Lingding Bay estuary, the largest of the three, is a funnel-shaped subaqueous delta. For convenience we will refer to this estuary as Lingding Bay.

Lingding Island divides the estuary into inner and outer Lingding Bay (Fig. 1C). The subaqueous topography of Lingding Bay is characterized by two deep troughs (East Trough and West Trough) between three shoals (East, Middle, and West Shoal) (Fig. 1C). West Trough is also known as Lingding Channel. The water depth in Lingding Bay varies between 2 m and 10 m in most areas and averages about 5 m. The deepest locations in the bay are deeper than 20 m^27^. Major cities around Lingding Bay include Hong Kong, Macao, Shenzhen, and Guangzhou (Fig. 1B). As Lingding Bay is the only sea access for the ports of Guangzhou and Shenzhen, its depth and topography are vitally important to these cities. Rampant urbanization is another ongoing influence on the natural processes of the Pearl River delta^27^.

### Water and sediment discharge

The China River Sediment Bulletin, issued by the Ministry of Water Resources of the People’s Republic of China, has provided annual statistics on the water and sediment discharge measured at the hydrological stations at Gaoyao (West River), Shijiao (North River), and Boluo (East River) since 1954 (Fig. 2)^25^. From 1955 to 2010, the annual average water and sediment discharges were 280 × 10^8^ m^3^/yr and 70 Mt/yr, respectively, and the West, North, and East rivers carried 88.3%, 8.2%, and 3.5% of the sediment, respectively.

The annual water and sediment discharges from 1955 to 2010 are shown in Fig. 2. From 1955 to 1983, water and sediment discharges increased substantially because of human activities such as aggravated soil erosion in the drainage basin^20^,^38^. However, the water and sediment discharges declined from 1994 to 2010. The Yantan Reservoir (completed in 1992) is one cause of the decline in sediment discharge after 1994, and the Longtan Reservoir (completed in 2007) is the main cause of the decline in sediment discharge after 2007. Sediment discharge decreased to 29.2 Mt/yr from 2006 to 2011^27^. Average sediment concentration decreased from 0.27 kg/m^3^ during 1980–2000 to 0.14 kg/m^3^ during 2000–2010.

### Coastal oceanography

Tidal regimes in the Pearl River estuary are mainly semi-diurnal (M_2_) and diurnal (K_1_) with a mean tidal range between 1.0 and 1.7 m (1.08 m at Hengmen and 1.69 m at Humen^39^). Along the shoreline, tidal currents are mainly reciprocal in the north-south direction whereas in the coastal waters, tidal currents rotate^35^. Analytical and numerical models have shown that river discharges and Kelvin waves interact to produce transverse gradients in the salinity density and sand content, then anticlockwise residual currents resulting from stronger flood currents in the east and stronger ebb currents in the west^40^,^41^.

The circulation outside the Pearl River delta is mainly forced by seasonal winds and interacts with the shelf circulation^42^,^43^,^44^. In response to the winter monsoon, mean surface currents are nearly south-westward outside the Pearl River estuary. In summer, when the south-westerly monsoon prevails in the South China Sea, the mean surface currents are nearly north-eastward over the northern South China Sea shelf (Fig. 1B).

## Methods

Single-beam bathymetric surveys were conducted in Lingding Bay in July 2012 and June 2013 with an HY1601 echo sounder (Wuxi Haiyang-Cal Tec Marine Technology Co.) with an accuracy of 1 cm ± 0.1% of water depth. The total length of 35 survey lines was 3,000 km (Fig. 5A). The navigation equipment used was the SF-3050 Global Positioning System (NavCom Technology Inc.), which has data positioning accuracy within ± 25 cm. HYPACK 2012 software (HYPACK, Inc.) was used for data acquisition, processing, and accuracy assessments.

We also used five bathymetric charts of the estuary, published by China Chart Publishing House in 1955, 1964, 1980, 2000, and 2010. The 1955, 1964, and 1980 charts were based on the Loran positioning system, with navigational accuracy of ~100 m, and the 2000 and 2010 charts were based on GPS, with navigational accuracy of ~10 m. Bathymetric data were acquired by echo sounder, yielding water depths with a precision of 1%. A submarine digital bathymetric model (DBM) with a resolution of 100 m × 100 m was constructed for the periods bounded by these dates. To calculate the annual bathymetry change rate (BCR) of different regions in the study area (Fig. 1C), we constructed a survey time model (STM) for each DBM. The digital bathymetry change model (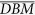) and survey time change model (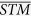) of different periods were calculated to obtain 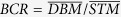 for each period. The model construction method was similar to that used in previous papers^12^,^27^,^45^,^46^, which used historical water depth data to study changes in estuarine bathymetry and geomorphology on a decadal time scale. The zero meter datum of the bathymetric charts is the lowest low water level, which is 1.7 m below mean sea level.

Maps in this paper were created with Surfer Version 7 (Golden Software, Golden, Colo., USA). Histograms and line charts were created with WPS Office, Version 2016 (Kingsoft Office Software, Hong Kong).

## Additional Information

**How to cite this article**: Wu, Z. Y. *et al*. Impact of human activities on subaqueous topographic change in Lingding Bay of the Pearl River estuary, China, during 1955–2013. *Sci. Rep.* **6**, 37742; doi: 10.1038/srep37742 (2016).

**Publisher's note:** Springer Nature remains neutral with regard to jurisdictional claims in published maps and institutional affiliations.

## Figures and Tables

**Table 1:** Land, tidal flat, and subaqueous areas in the Lingding Bay study area, 1955–2010.

Year		1955	1964	1980	2000	2010
Land area (km^2^)		476.87	477.67	502.40	688.08	710.17
Tidal flat area (km^2^)		215.06	282.10	290.06	170.93	159.29
Subaqueous area (km^2^)	0–2 m	270.56	235.76	229.27	173.16	200.98
	2–5 m	394.11	427.92	402.83	405.73	383.13
	5–10 m	293.40	236.70	236.29	205.84	162.38
	>10 m	52.22	42.33	41.28	58.75	86.16
	Subtotal	1,010.29	942.71	909.67	843.48	832.65
Subaqueous area (%)		59.35	55.37	53.44	49.54	48.92
Mean water depth (m)		4.40	4.25	4.20	4.50	4.60
Water volume (10^6^ m^3^)		4,445.30	4,006.50	3,820.60	3,795.70	3,830.20

**Table 2:** Sizes and changes of erosion and deposition areas, water volume, and land area in the Lingding Bay study area during 1955–2010.

Time period	1955–1964	1964–1980	1980–2000	2000–2010
Percentage of erosion area (%)	14.38	23.20	20.26	14.41
Percentage of deposition area (%)	39.48	28.83	26.70	31.44
BCR of erosion area (cm/yr)	−4.59	−3.10	−6.42	−16.00
BCR of deposition area (cm/yr)	6.48	4.70	4.80	9.35
Erosion + Deposition (%)	53.86	52.03	46.96	45.85
Water volume loss (10^6^ m^3^/yr)	11.23	12.24	22.14	39.24
Water volume gain (10^6^ m^3^/yr)	43.54	23.06	21.81	50.03
Water volume net gain (10^6^ m^3^/yr)	32.31	10.82	−0.33	10.79
Land area loss (km^2^)	244.75	394.86	344.83	245.26
Land area gain (km^2^)	671.95	490.69	454.43	535.11
Land area net gain (km^2^)	427.20	95.82	109.61	289.85
